# Differences in mortality in Switzerland by citizenship during the first and second COVID-19 waves: Analysis of death statistics

**DOI:** 10.3389/fpubh.2022.992122

**Published:** 2022-11-18

**Authors:** Tino Plümecke, Heiner Mikosch, Steffen Mohrenberg, Linda Supik, Isabelle Bartram, Nils Ellebrecht, Andrea zur Nieden, Laura Schnieder, Hannah Schönberger, Charlotte Schulze-Marmeling, Andreas Gutzeit

**Affiliations:** ^1^Independent Research Group SoSciBio, Institute of Sociology, University of Freiburg, Freiburg, Germany; ^2^Department of Health Sciences and Medicine, University of Lucerne, Lucerne, Switzerland; ^3^KOF Swiss Economic Institute, ETH Zürich, Zürich, Switzerland; ^4^Demo SCOPE AG, Adligenswil, Switzerland; ^5^Otto Suhr Institute of Political Science, Free University of Berlin, Berlin, Germany

**Keywords:** COVID-19, migration, Switzerland, death rates, vulnerable groups, age groups

## Abstract

**Background:**

Early in the COVID-19 pandemic, it became apparent that members of marginalized populations and immigrants were also at risk of being hospitalized and dying more frequently from COVID-19. To examine how the pandemic affected underserved and marginalized populations, we analyzed data on changes in the number of deaths among people with and without Swiss citizenship during the first and second SARS-CoV-2 waves.

**Method:**

We analyzed the annual number of deaths from the Swiss Federal Statistical Office from 2015 to 2020, and weekly data from January 2020 to May 2021 on deaths of permanent residents with and without Swiss citizenship, and we differentiated the data through subdivision into age groups.

**Results:**

People without Swiss citizenship show a higher increase in the number of deaths in 2020 than those who were Swiss citizens. The increase in deaths compared to the previous year was almost twice as high for people without Swiss citizenship (21.8%) as for those with it (11.4%). The breakdown by age group indicates that among people between the ages of 64 and 75, those without Swiss citizenship exhibited an increase in mortality (21.6%) that was four times higher than that for people with Swiss citizenship (4.7%).

**Conclusion:**

This study confirms that a highly specialized health care system, as is found in Switzerland, does not sufficiently guarantee that all parts of the population will be equally protected in a health crisis such as COVID-19.

## Introduction

COVID-19 has caused a sizeable global outbreak and a fundamental public health issue but the pandemic did not affect everyone to the same extent. Early on, it became apparent in many studies that various socioeconomic factors cause people to be affected very differently (1–8).

A recent systematic review of 52 studies found moderate to solid evidence that in the US, especially African American/Black and Hispanic populations experience a disproportionate burden of SARS-CoV-2 infections and COVID-19-related mortality (9). However, analyses in the United States always face the problem that the health care system is considered particularly expensive and inefficient and that, unlike in most European countries, there is no guaranteed care for the entire population (10).

Switzerland is considered to have one of the best health care systems in the world, which reaches the entire population in essentially the same manner and scope (11). A major study has shown that Switzerland ranks among the top three healthcare systems in the world regarding access to healthcare and successful treatment of diseases (12). The life expectancy in Switzerland (82.8 years) is the highest in Europe after Iceland, and healthy life expectancy is several years above the EU average (11).

Despite this good health care system, it is difficult in Switzerland to uncover differences in the impact of COVID-19 on marginalized populations and to compare them to data from the United States or other countries. This is because data on hospitalized patients' ethnic or racial identity or immigrant status are not recorded regularly in Switzerland. However, in Switzerland death data can be linked to citizenship data, allowing a subdivision into people with and without Swiss passports. People without Swiss citizenship make up about 26% of the permanent resident population in Switzerland (27% of men and 24% of women), with Italy, Germany, Portugal, France, Kosovo, Spain, and Turkey as the main countries of origin. They include immigrants and—due to high naturalization barriers—the children and grandchildren of immigrants who are born in Switzerland.

If one considers only the effectiveness of the health care system, the risk of dying from COVID-19 in Switzerland could be expected to be better managed than in countries without guaranteed access to health care services. Furthermore, differences between various segments of the population in infection rates, hospitalization, and mortality should be small. To address how the pandemic affects the two population segments of people with and without Swiss citizenship, we compared the increase in the number of deaths in the first year of the pandemic. With the data provided by the death statistics, only limited conclusions can be drawn about the effects of social inequality and discrimination on health variables. Nevertheless, the differences in the number of deaths make it possible to spotlight one area of concern.

## Methods

We analyzed the death statistics of people with and without Swiss citizenship provided by the Swiss Federal Statistical Office. We used weekly deaths from January 2020 to May 2021 (the first two infection waves where general vaccination was not yet available) as well as annual death numbers from 2015 to 2020 (1969–2020 in the Supplementary material), disaggregated by age groups and citizenship status. The number of deaths without Swiss citizenship is calculated as the difference between the total number of deaths and the number of deaths with Swiss citizenship. Hence, the deaths among those without Swiss citizenship also include persons who died without any citizenship (stateless persons) and deaths with status “unknown” regarding their citizenship. Swiss authorities record data such as citizenship for all residents of Switzerland who have a residence title based on the Ordinance on Civil Status of the Federal Council (*Zivilstandsverordnung*). In the event of a death, these data are checked by the authorities, which makes it practically impossible—according to the Swiss Federal Statistical Office—for a person with Swiss citizenship to be listed in the official death statistics with the citizenship status “unknown.” In 2019 and 2020, only one deceased person was “stateless,” and 27 (2019) and 73 (2020) deaths were recorded as “unknown” regarding their citizenship status.

In this article, we first analyze the total number of deaths (13) in a year-to-year comparison (see also Supplementary Figure 1) as well as weekly data from 2020 and the first weeks of 2021 relative to the death data in 2019. Second, we compare the increase in mortality (deaths per 100,000 persons) for all deaths in 2020 and for the age groups for both segments of the population (14). This separate mortality calculation is necessary, since in Switzerland demographic changes are mainly caused by younger people added through births and immigration, while it is mainly older people who die from COVID-19. In order to compare the increase in mortality in different age groups, we show the difference between the measured value for 2020 relative to the mortality that would have been expected for 2020 based on the extrapolation of a citizenship and age group-specific linear trend (using linear regression, the confidence level was set at 95%). We consider that a linear trend based on the last 5 years describes relatively well the development of the observed data of the number of deaths for most age groups, both with and without Swiss citizenship. Therefore, we believe that a linear trend can also provide a realistic expected value for 2020 and offers an advantage over the repeatedly used method of setting the expected mortality for a year to the average mortality of the previous 5 years. In particular, the method we use accounts for the group-specific trends in mortality over time, which is especially relevant for age groups without Swiss citizenship, as can be inferred from Supplementary Figure 1.

## Results

### Increase in the number of deaths in 2020

In 2020, the overall number of deaths in Switzerland increased by 12.4% compared to the previous year. The increase in deaths in the first year of the COVID-19 pandemic is thus the highest annual increase since 1918 and more than double the highest annual growth measured in the previous 10 years (15). Of course, the number of deaths varies yearly, mainly due to severe influenza epidemics or heat waves during the summer. Nevertheless, the number of 8,415 additional deaths in 2020 compared to the previous year is close to the 9,294 COVID-19-related deaths documented by the Federal Statistical Office (16).

**Figure 1:**
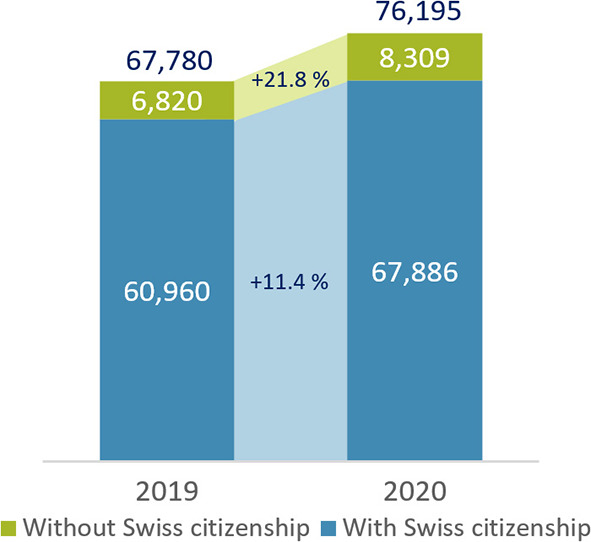
Total number of deaths in Switzerland in 2019 and 2020. Data source: Swiss Federal Statistical Office (13).

Figure 1 shows the differences in the increase in the number of deaths among people with Swiss and without Swiss citizenship. In 2020, the increase in the total number of deaths compared to the previous year was almost twice as high for people without Swiss citizenship (21.8%) as for those who were Swiss citizens (11.4%). It should be noted that the population with Swiss citizenship grew by 0.45% compared to 1.6% for people without. However, a correction to the population growth should be made with caution (see material and methods section).

It is essential to note that among people living in Switzerland without Swiss citizenship, the proportion of elderly people is lower than among people with Swiss citizenship. For example, people aged 80 and older account for 6.4% of people with a Swiss passport, while the proportion of those without Swiss citizenship among those 80 or older is only 2.1% (17). This means that one should suppose that during the pandemic—under otherwise identical conditions—the increase in deaths among persons with Swiss citizenship would exceed that among persons without Swiss citizenship. Our data analysis, by contrast, shows the opposite.

### Differences during waves of COVID-19

**Figure 2:**
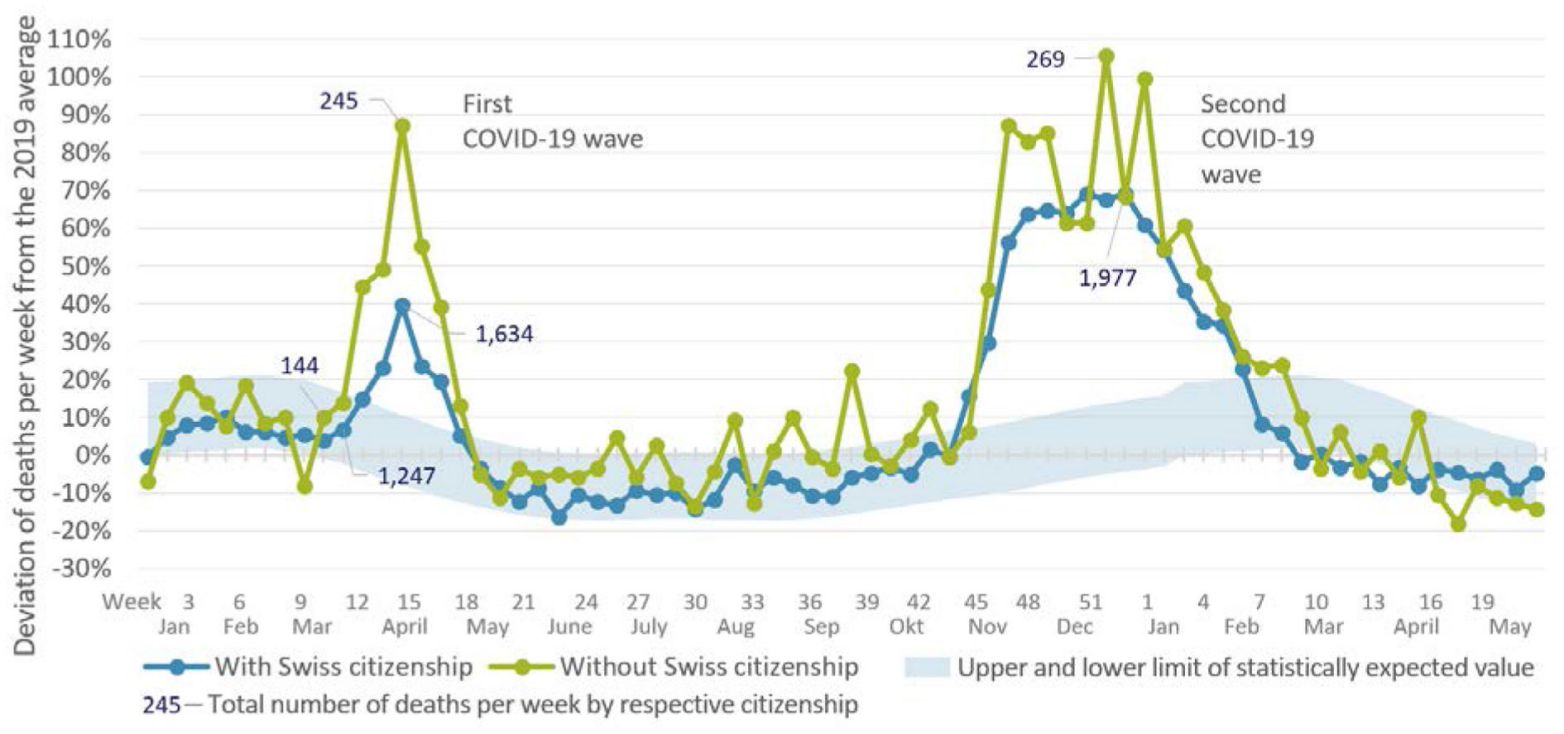
Changes in weekly deaths in Switzerland from 2020 to May 2021 relative to the average number of deaths per week in the previous year (1,169 people with Swiss citizenship and 131 people without Swiss citizenship). The light blue band represents the number of deaths expected within the framework of random fluctuations, based on the development of the case numbers of all those who died in Switzerland in the previous 10 years. Source: Unpublished data from the Swiss Federal Statistical Office.

Figure 2 compares the weekly data on deaths in the two population segments during the first and second infection waves. The level differences are plotted based on the ratio to the respective number of deaths in 2019. The number of deaths of people without Swiss citizenship showed a greater increase in both waves than for those with Swiss citizenship.

At the peaks of both waves, about twice as many people without Swiss citizenship died than would have been expected based on the data before the COVID-19 pandemic (first wave: week 14, 245 deaths; second wave: week 52, 269 deaths). In addition, the number of people who died without Swiss citizenship increased in the first wave even before the increase in deaths in the total population.

### Different mortalities across age groups

**Figure 3:**
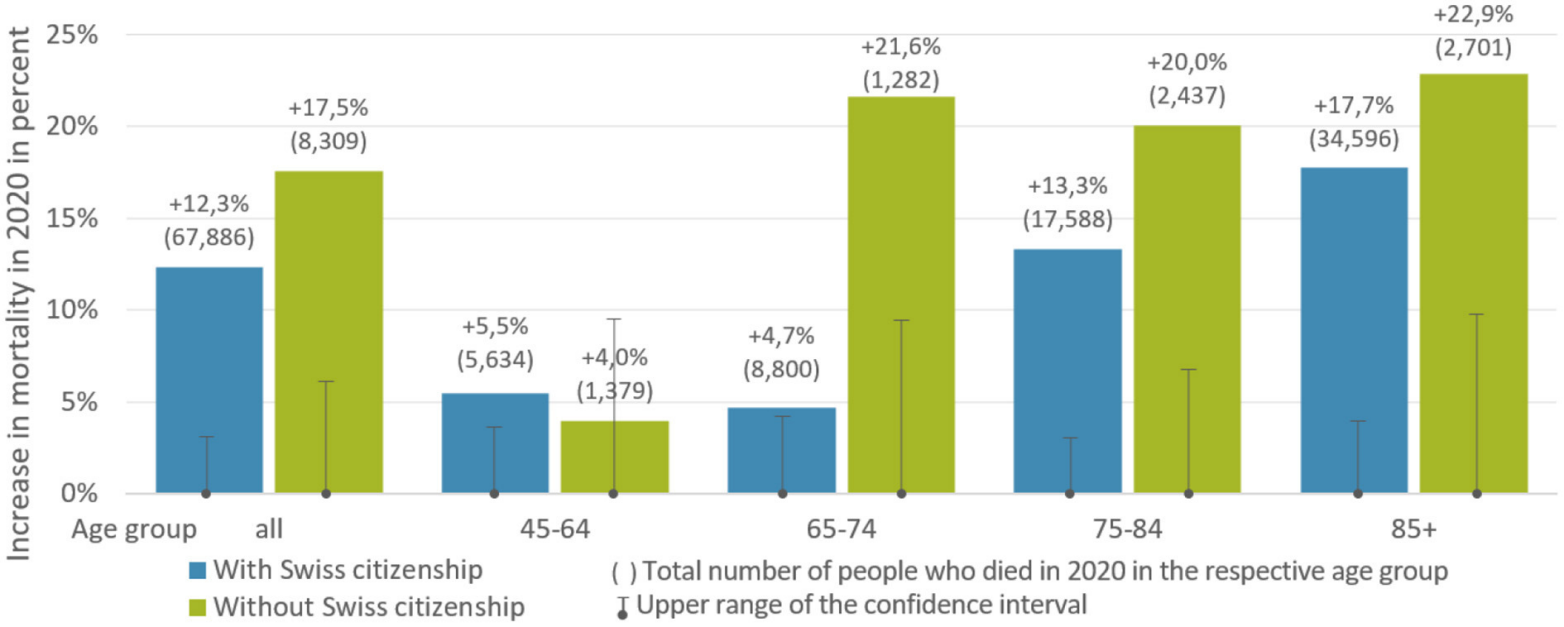
Citizenship and age group-specific increase in mortality in 2020 relative to the mortality expected for 2020 based on the extrapolation of a respective group-specific linear trend over the years 2015–2019. Data source: Death statistics of the Swiss Federal Statistical Office (18).

Further differences in the increase become apparent in Figure 3, where we differentiate by age group. The figure shows, for each population segment, the percentage increase in mortality in 2020 relative to the mortality that would have been expected for 2020 based on the extrapolation of a linear trend over the years 2015–2019 (see Supplementary material for more details). The upper bound of the confidence interval for each group is shown in the small bars as the percent change in the distribution relative to the mean expected number of deaths for 2020. As can be seen from the figure, this percentage change is positive for all considered groups, i.e., the mortality in the year 2020 shows a greater increase than expected in all groups. For persons without Swiss citizenship, however, the increase is much more substantial than for those with Swiss citizenship (+17.5 vs. +12.3%).

When distinguishing between the three age groups of those over 64, further differences between persons with and without Swiss citizenship become visible. For example, the actual number of deaths in 2020 in the age group of 65–74 without Swiss citizenship is significantly higher than the expected mortality for the year 2020 and more than four times higher than for persons with Swiss citizenship (+21.6 vs. +4.7%). This is noteworthy because, among persons with Swiss citizenship, an increase was observed particularly in the age group of 75–84 years old and even more strongly in the age group of 85 years and older. Not presented are the years from 0 to 44, since they are not regarded as highly affected by increased mortality associated with COVID-19 (19). In our analysis, these age groups (0–44) also do not differ significantly in mortality. In addition, because of the small number of deaths in these age groups, the results that can be obtained have only limited statistical validity. These difficulties with small death numbers are still visible in the graph for the age group of 45–64. This group's annual number of deaths is comparatively low, while the variations are relatively high. Nevertheless, there was an increase of mortality in both groups in 2020 compared to the mortality expected in the same year.

## Discussion

This analysis of the death statistics during the first two waves of the COVID-19 pandemic revealed a substantial increase in the number of deaths among people with and without Swiss citizenship in Switzerland. Among people with Swiss citizenship, an increase in deaths of 11.4% over the year before the pandemic was recorded, whereas the proportion of deaths among people without Swiss citizenship increased by 21.8% in the same period. Further differentiation—by weekly data and by age groups—shows that the increased number of deaths among people without Swiss citizenship started 1 week before that of people with Swiss citizenship and shows a much larger increase compared to the year before. In addition, the comparison of the mortality of age groups shows a particular vulnerability of people without Swiss citizenship in early retirement age (65–74). However, our findings from the analysis of the death statistics of people with and without Swiss citizenship should not be understood as limited to these segments of the population. Instead, they point to a broader area of concern.

Numerous studies worldwide have shown an increased risk for members of marginalized and underserved communities and immigrants dying from COVID-19 compared to the majority population. This is usually interpreted as resulting from inadequate medical care in the respective health systems, a generally high level of inequality in society that also leads to differing comorbidities, and differences in the level of trust in the medical profession and the health sector in general (9, 20, 21). The question that arises is whether these reasons are also the cause of the differences in mortality between people with and without Swiss citizenship presented in this study.

The Swiss healthcare system is considered to be one of the best healthcare systems in the world, with a high degree of specialization and low-threshold access to health care for the entire population (12). It is necessary to mention that the cost of this effort is relatively high. In 2019, Switzerland invested 11.3% of gross domestic product (GDP) in healthcare spending as a percentage of national income, whereas the average of all OECD states was nearly 9% in the same year (22). Despite the high quality of the health care infrastructure, our analyses show that the COVID-19 pandemic affected people without Swiss citizenship substantially more.

Based on international research on health inequities, differences in the increases in deaths associated with citizenship can be expected to correlate with socioeconomic disparities (23, 24). Income and wealth inequalities, household crowding, occupations with direct customer contact or in labor-intensive or physically demanding areas, and reliance on public transport may all increase the risk of infection (25, 26). Socioeconomic status also correlates with a higher risk for a wide range of diseases, such as hypertension, hyperlipidemia, brain ischemia, alcoholism, nicotine addiction, and obesity (27) that are also associated with severe disease progression in COVID-19 infection. In addition, economic segregation and experiences of discrimination can significantly affect health status and disease risks (28–30).

The measured disparity in the effect of COVID-19 on different segments of the population in Switzerland can be attributed to comparatively high inequality in income, besides the factors just mentioned. Mortality from COVID-19 is closely related to income inequality, for example, as quantified in the Gini coefficient (31, 32). When considering economic equality, Switzerland performs relatively poorly compared to other European countries. The prevailing paradigm that highly specialized medicine and high total spending lead to better care for the entire population must be questioned in light of the data presented here. Social equity may be more relevant than the degree of specialization and overall health care spending (33). This means that in addition to classic risk factors, social determinants should be considered as the more relevant factors for improving and maintaining health (34). We advocate for closer cooperation between public health and prevention efforts, in addition to a good health care infrastructure, as the most vulnerable parts of society will again be at high risk of insufficient protection and support during future health crises.

### Limitations

The health data available in Switzerland to date do not adequately reflect the impact of social inequality and structural disadvantages, with the consequence that health inequity and support needs may be difficult to identify. It should be noted that it is not the possession or non-possession of citizenship that causes the differences in death rates. It is not migrants *per se* who die at a higher rate; what is significant here is that social and health inequalities have different effects on different segments of the population.

To determine health inequity, the differentiation between people with or without Swiss citizenship can only serve as an imprecise proxy for identifying severe mortality differences in underserved or marginalized populations. It is also important to note that we cannot use the relatively crude category of citizenship to represent diversity within individual population groups. This is because people with a migration history who hold a Swiss passport yet may be particularly vulnerable are not included in the category of people without Swiss citizenship, while foreign professionals and managers, for example, are included, even though most of them do not face increased vulnerability. In the future, it would be useful to look at how socioeconomic differences affect mortality. Unfortunately, this data is not systematically recorded in death statistics in Switzerland.

In addition, only data on all deaths, that is, without differentiating causes of death, are available for Switzerland. Therefore, processes unrelated to the COVID-19 pandemic but affecting different segments of the population differently could also play a role. The Swiss Federal Office of Public Health (FOPH) has made an additional record of COVID-19-related deaths that also asks about the “nationality” of those who died. However, in 51% of the cases, no information was given on nationality. A valid analysis is therefore not possible with these incomplete data.

Additional possible limitations exist in our choice of the prediction method for the expected number of deaths in the respective age groups and the possession and non-possession of Swiss citizenship. The 5-year period chosen for the trend calculation seemed to us to be the most reasonable based on the developments since 1969 (see Supplementary material). Since the differences between predicted and observed values were highly significant, the choice of a slightly different period (e.g., 3 or 7 years) would also not produce serious deviations in the results.

Biases in the analysis are also to be expected due to the different age structures of the population segments with or without Swiss citizenship. The healthy migrant effect can also lead to a potential distortion. However, both of these factors are likely to result in a lower number of deaths among persons without Swiss citizenship, and thus to have reduced rather than increased the differences in death rates presented here.

## Conclusion

Switzerland is considered to have one of the highest quality healthcare systems in the world, providing a broad access to health care for the whole population. Nonetheless, in the first two COVID-19 waves, there were significant differences in the death rates of people with or without Swiss citizenship. In addition to the known risk factors of age and preexisting conditions, further vulnerabilities are also associated with an increased risk and indicate a corresponding need for social protection and support. To reduce health risks and increased mortality in the future, socioeconomic risks and social equity should be given much greater consideration than they are now.

## Data availability statement

The original contributions presented in the study are included in the article/Supplementary material, further inquiries can be directed to the corresponding author.

## Author contributions

Material preparation, data collection, and analysis were performed by TP, LS, SM, and HM. Those responsible for the first draft of the manuscript and editing the manuscript were TP and AG. The data analysis and presentation were conducted by TP, SM, HM, HS, NE, and CS-M. All authors contributed to the study conception and design, commented multiple times on previous manuscript versions, read, and approved the submitted version.

## Funding

The Independent Research Group SoSciBio was funded by the German Federal Ministry of Education and Research, FKZ 01GP1790.

## Conflict of interest

Author SM was employed by Demo SCOPE AG. The remaining authors declare that the research was conducted in the absence of any commercial or financial relationships that could be construed as a potential conflict of interest.

## Publisher's note

All claims expressed in this article are solely those of the authors and do not necessarily represent those of their affiliated organizations, or those of the publisher, the editors and the reviewers. Any product that may be evaluated in this article, or claim that may be made by its manufacturer, is not guaranteed or endorsed by the publisher.

## Supplementary material

Supplementary Figure 1 Observed annual number of deaths from 1969 to 2020 broken down by citizenship status and age groups (1969–2020 in black, blue dots for 2020). For each of the subpopulations, we calculated a linear trend based on the annual numbers of deaths from 2015 to 2019 (red lines) and, by extrapolating this trend 1 year into future, a mean expected number of deaths for 2020 (red dots) together with a probability range (vertical red lines). For the calculation of the probability range for each subpopulation, we assume that the number of deaths of each year *t* (e.g., the year 2020) follows a Poisson distribution, with the rate parameter being equal to the respective mean expected death number for year *t*. The lower bound and the upper bound of the probability range are the 2.5th percentile and the 97.5th percentile of the Poisson distribution, respectively. It should be noted that due to the very different ranges of observed y-values across the subfigures, different scaling and a shortening of the y-axes was necessary.
