# Supplementary material for: Differences in mortality in Switzerland by citizenship during the first and second COVID-19 waves: Analysis of death statistics
Source: Front Public Health. 2022 Nov 18;10:992122. doi: 10.3389/fpubh.2022.992122 (PMC9716092; doi:10.3389/fpubh.2022.992122)
Supplement: Supplementary Figure 1 — Observed annual number of deaths from 1969 to 2020 broken down by citizenship status and age groups (1969–2020 in black, blue dots for 2020). For each of the subpopulations, we calculated a linear trend based on the annual numbers of deaths from 2015 to 2019 (red lines) and, by extrapolating this trend 1 year into future, a mean expected number of deaths for 2020 (red dots) together with a probability range (vertical red lines). For the calculation of the probability range for each subpopulation, we assume that the number of deaths of each year t (e.g., the year 2020) follows a Poisson distribution, with the rate parameter being equal to the respective mean expected death number for year t. The lower bound and the upper bound of the probability range are the 2.5th percentile and the 97.5th percentile of the Poisson distribution, respectively. It should be noted that due to the very different ranges of observed y-values across the subfigures, different scaling and a shortening of the y-axes was necessary. [file Image_1.pdf]

Supplementary Material

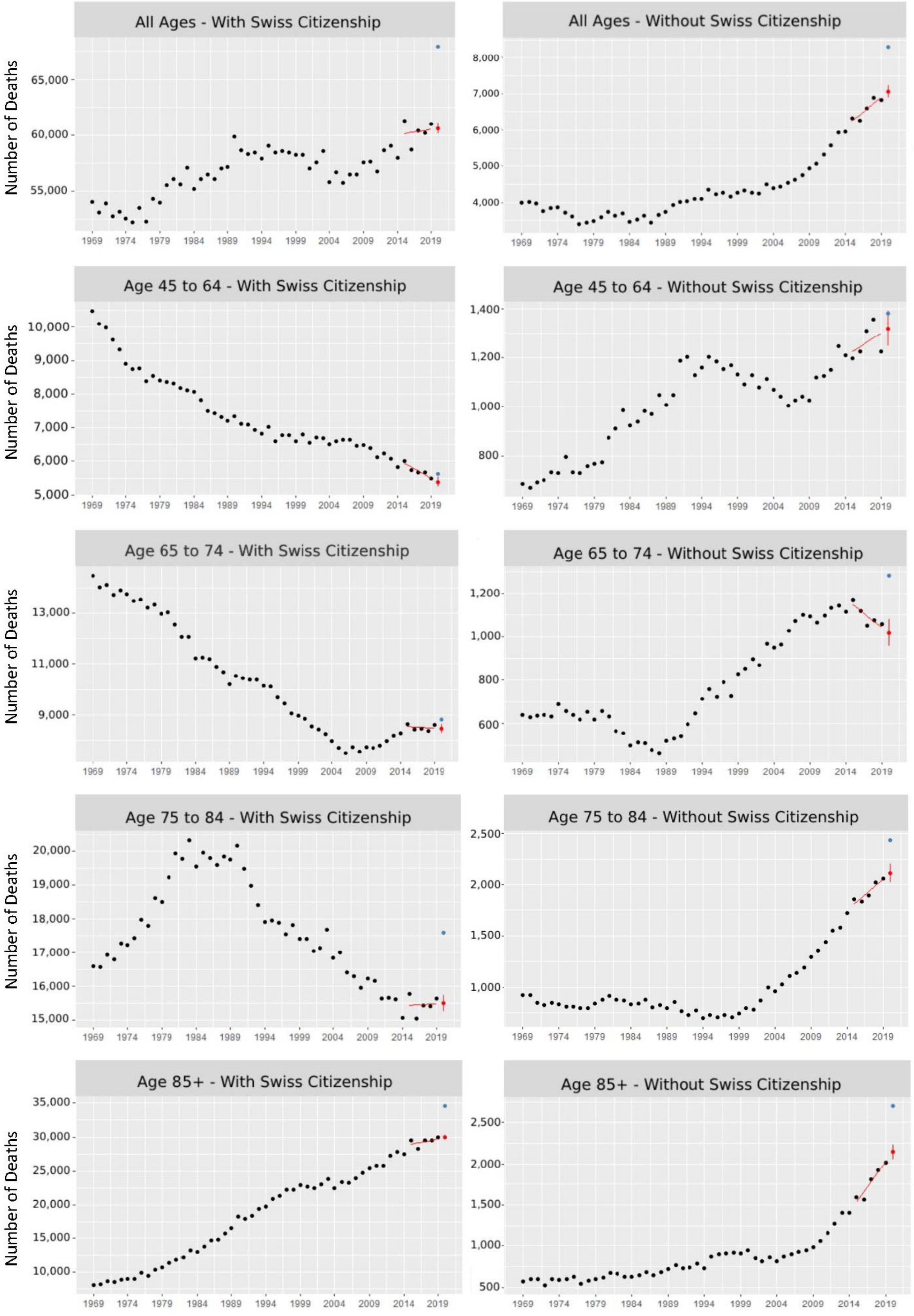

Supplementary Figure 1 | Observed annual number of deaths from 1969 to 2020 broken down by citizenship status and age groups (1969 to 2020 in black, blue dots for 2020). For each of the subpopulations, we calculated a linear trend based on the annual numbers of deaths from 2015 to 2019 (red lines) and, by extrapolating this trend one year into future, a mean expected number of deaths for 2020 (red dots) together with a probability range (vertical red lines). For the calculation of the probability range for each subpopulation, we assumed that the number of deaths of each year *t* (e.g., the year 2020) follows a Poisson distribution, with the rate parameter being equal to the respective mean expected death number for year *t*. The lower bound and the upper bound of the probability range are the 2.5th percentile and the 97.5th percentile of the Poisson distribution, respectively. It should be noted that due to the very different ranges of observed y-values across the subfigures, different scaling and a shortening of the y-axes was necessary.
